# Wastewater Treatment by a Polymeric Bioflocculant and Iron Nanoparticles Synthesized from a Bioflocculant

**DOI:** 10.3390/polym12071618

**Published:** 2020-07-21

**Authors:** Nkosinathi Goodman Dlamini, Albertus Kotze Basson, Rajasekhar VSR Pullabhotla

**Affiliations:** 1 Department of Biochemistry and Microbiology, University of Zululand, Private Bag X1001, KwaDlangezwa 3886, South Africa; BassonA@unizulu.ac.za; 2 Department of Chemistry, University of Zululand, Private Bag X1001, KwaDlangezwa 3886, South Africa

**Keywords:** biosafety, flocculation, removal efficiency, wastewater

## Abstract

Wastewater remains a global challenge. Various methods have been used in wastewater treatment, including flocculation. The aim of this study was to synthesize iron nanoparticles (FeNPs) using a polymeric bioflocculant and to evaluate its efficacy in the removal of pollutants in wastewater. A comparison between the efficiencies of the bioflocculant and iron nanoparticles was investigated. A scanning electron microscope (SEM) equipped with an energy-dispersive X-ray analyzer (EDX) and Fourier transform-infrared (FT-IR) spectroscopy were used to characterize the material. SEM-EDX analysis revealed the presence of elements such as O and C that were abundant in both samples, while FT-IR studies showed the presence of functional groups such as hydroxyl (–OH) and amine (–NH_2_). Fe nanoparticles showed the best flocculation activity (FA) at 0.4 mg/mL dosage as opposed to that of the bioflocculant, which displayed the highest flocculation activity at 0.8 mg/mL, and both samples were found to be cation-dependent. When evaluated for heat stability and pH stability, FeNPs were found thermostable with 86% FA at 100 °C, while an alkaline pH of 11 favored FA with 93%. The bioflocculant flocculated poorly at high temperature and was found effective mostly at a pH of 7 with over 90% FA. FeNPs effectively removed BOD (biochemical oxygen demand) and COD (chemical oxygen demand) in all two wastewater samples from coal mine water and Mzingazi River water. Cytotoxicity results showed both FeNPs and the bioflocculant as nontoxic at concentrations up to 50 µL.

## 1. Introduction

Approximately 90% of wastewater is discharged untreated into water bodies in developing countries (Corcoran) [1]. The aquatic ecosystem is threatened by this as edible and drinkable water become contaminated [2]. Colloids are heterogeneous matter characterized by kinetically non-labile and thermodynamically instable characteristics. Colloids, organic, and inorganic pollutants in water are a major concern of this era. Colloids have a tendency of not settling under gravity in a solution [3]. Both organic and inorganic hazardous pollutants, including derivatives of phenols and dyes released from different industries, have turned out to be a global problem [4,5]. Textile industries are one of the largest sources that are contributing to the pollution of water. This is due to the application of different chemicals throughout the textile processing [6,7]. Untreated effluent discharge from the textile processing results in highly toxic wastewater [8]. This effluent contains high levels of chemical oxygen demand (COD) and biochemical oxygen demand (BOD) and is highly turbid. The release of this untreated effluent to sea, lakes, or rivers affect the environment badly [9]. In developing countries, close to 10% of the population dies due to waterborne infections as well as cancer caused by untreated industrial effluents in water [10]. Hence, treatment and removal of the pollutants that are present in water bodies are necessary, though it is never an easy task.

Several techniques have been employed to treat the effluents and to remove toxic compounds from the water [10,11]. The methods include constructed wetlands, membrane filtration, hybrid ion exchange materials and electrocoagulation, etc. All these water treatment technologies play a substantial role in the treatment of effluents from industries. However, the major downside of these techniques is that they are either very expensive or produce immense amounts of sludge [12]. 

Of late, secondary metabolites (bioflocculants) produced by microorganisms during growth are viewed as the possible solution to water treatment. These flocculants are favored due to their environmental friendliness, biodegradability, and nontoxicity [11], and they cause no environmental harm and can remove heavy metals from wastewater [13]. Ugbenyen and Okoh [14] stated that chemicals that stimulate flocculation by aggregation of colloids and other suspended particles, forming a floc, are called flocculants. Both organic and inorganic flocculants have been used in the purification of water in various industries. This includes organic synthetic polymers, inorganic aluminium, and ferric salt [15]. Natural flocculants have also been used in various downstream processes such as treatment of wastewater, purification of potable water, and fermentation and food industries [16]. Commonly, the flocculants categories are: synthetic organic flocculants, which include polyacrylamide derivatives; inorganic flocculants, which include polyaluminium chloride; and naturally occurring flocculants, which include the secondary secretion (bioflocculants) from microorganisms [17]. 

Flocculants of chemical nature have been used widely in the process of flocculation due to the cost effectiveness and flocculating efficiency [18]. Nonetheless, some environmental and health concerns have been raised through their usage due to the monomers of these flocculants being reported as toxic to humans, and aluminium salts being associated with Alzheimer’s disease [11,19]. Therefore, researchers in the world have shifted focus in the application of these biodegradable, environmentally friendly flocculants to replace chemically synthesized flocculants. Despite all these interesting properties of biodegradability and environmental-friendliness, natural flocculants have the disadvantages of low shelf life, are very expensive to produce, have low yield, and have minimal flocculation activity [18]. Therefore, to overcome these shortcomings, we investigate the application of bioflocculant-synthesized nanoparticles in comparison to chemical synthetic flocculant (ferric chloride) and bioflocculant.

Hence, in the present study, we report the synthesis of iron nanoparticles using a polymeric bioflocculant, and its application in wastewater treatment in comparison to a bioflocculant and biosafety evaluation.

## 2. Materials and Methods

### 2.1. Production Medium Chemicals

All reagents for production media used were obtained from Sigma-Aldrich (St. Louis, MO, USA). The standard production medium as described by Zhang, et al. [20] was followed. A litre of the filtered sea water was used together with the following reagents: glucose (20.0 g), KH_2_PO_4_ (2.0 g), K_2_HPO_4_ (5.0 g), (NH_4_)_2_SO_4_ (0.2 g), NaCl (0.1 g), CH_4_N_2_O (0.5 g), MgSO_4_ (0.2 g), and yeast extract (0.5 g).

### 2.2. Extraction and Purification of the Bioflocculant

The bacteria used were previously isolated from the sediment sample from Sodwana Bay in the Province of KwaZulu-Natal in South Africa (28°450′ S 31°540′ E) and identified as *Alcaligenes faecalis* HCB2 [11]. Bioflocculant extraction was achieved following a method as described by Dlamini et al. [21]. Firstly, 1 L of the production medium was prepared and autoclaved at 121 °C for 15 min. Subsequently, 1% in (50 mL) inoculum was added and the medium incubated in a shaker at 165 rpm for 72 h at 30 °C, and after incubation, the medium was centrifuged at 8000 rpm at 4 °C for 30 min. This was done in order to remove cells and insoluble substances. The supernatant was transferred into a clean container and 1 L of distilled water and 2 L of ethanol were added to the supernatant, agitated, and the solution was stored at 4 °C for 12 h. Later, the precipitate formed was vacuum-dried and 100 mL of distilled water was added. A mixture of chloroform and *n*-butyl (5:2 *v*/*v*) was also added and the mixture was left to stand for 12 h at room temperature [21].

### 2.3. Synthesis of the Iron Nanoparticles (FeNPs)

To synthesize iron nanoparticles, a green-approach method was adopted [22]. A metal precursor for the synthesis of iron nanoparticles (FeNPs) used was iron sulphate (FeSO_4_). Briefly, 0.5 g of pure bioflocculant was dissolved in 0.2 M (FeSO_4_), and to prevent agglomeration of nanoparticles, 10 mL of 5.0 M sodium hydroxide (NaOH) solution was added. The mixture was left overnight at room temperature and nanoparticles formation was confirmed by physical observation, i.e., color change and characterization. Subsequently, the mixture was centrifuged at 5000 rpm at 4 °C for 15 min to harvest the synthesized nanoparticles and the resulting precipitate was vacuum-dried at 25 °C for 24 h [22].

### 2.4. Characterization of the Bioflocculant and Iron Nanoparticles

#### 2.4.1. Morphology and Element Analysis

A scanning electron microscope (SEM, JEOL, USA, Inc., Peabody, MA, USA) and energy-dispersive X-ray spectroscopy (EDX, JEOL, USA, Inc., Peabody, MA, USA) were used to evaluate morphology and elements in FeNPs and the bioflocculant.

#### 2.4.2. Functional Groups Analysis

Fourier transform-infrared (FT-IR, Bruker, Gauteng, South Africa) spectroscopy was used to identify and confirm the functional groups present in FeNPs and the bioflocculant (Tensor 27, Bruker FT-IR spectrophotometer, Bruker, Gauteng, South Africa).

### 2.5. Determination of Flocculation Activity

The process in which mediation of flocculants is achieved in the presence of microorganisms or biodegradable macromolecular flocculants released by microorganisms is called bioflocculation.

Kaolin clay was used as the test material in this study; 4.0 g in 1 litre distilled water was prepared. Kaolin clay solution (50 mL) was added into a 250 mL conical flask, and thereafter, 2.0 mL (0.2 mg/mL) solution of the bioflocculant or iron nanoparticles was added and 3.0 mL CaCl_2_ (1.0 g/L) solution was also added. The mixtures were shaken for 1 min and transferred to 100 mL graduated measuring cylinders. The mixture was left to stand for 5 min before the supernatant was taken for analysis [23]. The following equation was used to calculate the flocculation activity:

Flocculation activity

(1) $$FA\;\%=\frac{\left[A-B\right]}{A}\times 100$$

where *A* is the optical density of a control at 550 nm and *B* is the optical density of a sample at 550 nm. The flocculation mechanism for the bioflocculant is bridging [11].

### 2.6. Optimization of the Flocculation Efficiency of the Bioflocculant and FeNPs

#### 2.6.1. Evaluation of Flocculation Activity of the Bioflocculant and FeNPs

To evaluate the most effective dosage, different concentrations were prepared (0.2, 0.4, 0.6, and 0.8 mg/mL) by dissolving the bioflocculant and FeNPs in distilled water to obtain respective concentrations. A liter of kaolin solution was prepared using distilled water (4 g/L), after which, 100 mL of kaolin solution, 2 mL of the bioflocculant or FeNPs, and 3 mL of 1% CaCl_2_ were transferred into a 300 mL conical flask. The mixture was vigorously shaken for 1 min before being transferred into a measuring cylinder (100 mL) and allowed to settle for 5 min at room temperature. This procedure was also followed for the control, where 2 mL of nanoparticles were replaced by 2 mL distilled water. The clear top layer of the supernatant was pipetted into a cuvette to determine the flocculation activity. A UV-Visible spectrophotometer was used to measure the optical density (OD_550nm_). All experiments were conducted in triplicates. Equation (1) above was used to calculate the flocculation activity. The most effective dosage was used for the subsequent experiment and kaolin clay used as test material.

#### 2.6.2. Effect of Cations on Flocculating Activity

Different salts were used to ascertain cation effect on flocculation activity, solutions were used to replace 1% CaCl_2_, and the salts used were comprised of monovalent (LiCl and NaCl), divalent (MgCl_2_ and CaCl_2_), and trivalent (FeCl_3_) at the same concentration. The control was maintained without cations. To measure the flocculating activity, the above procedure was used to evaluate cation effect on flocculation activity.

#### 2.6.3. Effect of pH and Temperature on Flocculating Activity

A solution of NaOH (1.0 M) or HCl (1.0 M) was used whenever necessary to adjust pH in a range (3 to 11). The flocculation activity was assessed using the previously described method. Both the bioflocculant and FeNPs were subjected to high temperatures (50–100 °C) in a water bath for 30 min to determine thermostability, after which the flocculation activity was calculated using the method described above.

### 2.7. Wastewater Treatment

To assess removal efficiency (RE), coal mine wastewater and Mzingazi River water samples were collected and autoclaved at 121 °C for 15 min to ensure that no microorganisms were present to interfere with experimentation. The samples were collected from Tendele Coal Mine and Mzingazi River in KwaZulu Natal, RSA. Following the method described by Maliehe, Basson, and Dlamini [11], COD and BOD removal was evaluated. A UV-Vis spectrophotometer Pharo 300 Spectroquant^®^ was used at 680 nm for the RE measurement. The removal efficiency (RE) of the pollutants was calculated by the following equation:

(2) $$RE\;\left(\%\right)=\frac{C_{i}-C_{f}}{C_{i}}\times 100$$

where: *C_i_* is the initial value before treatment with the bioflocculant and nanoparticles and *C_f_* is the value after treatment.

### 2.8. Cytotoxicity of the Bioflocculant and Iron Nanoparticles

A method described by Daniels and Singh [24] was adopted to evaluate cytotoxicity of the bioflocculant and nanoparticles using human embryonic kidney (HEK 293) and breast cancer cells (MCF-7). Cells with cell suspensions of 1 × 10^5^ cells/mL concentrations were platted on 96-well-plate. Using a tenfold serial dilution method, the cells were seeded with different concentrations of nanoparticles (25–100 µg/µL). After 48 h of incubation, media containing 1% of fetal bovine serum (FBS) were used for the administration of nanoparticles and the plates were returned to the incubator for 48 h. To ascertain cell viability, tetrazolium salt (Sigma) was added as an indicator after 48 h of incubation. Then, 15 μL of MTT (5 mg/mL) in phosphate-buffered saline (PBS) was added to each well and incubated at 37 °C for 4 h. After sucking off from the wells, the medium with MTT and the formed formazan crystals were dissolved in 100 μL of dimethyl sulfoxide (DMSO). The optical density of the solutions was measured at 570 nm using a microplate reader [24].

The % cell inhibition was determined using the following formula:

Cell viability $\left(\%\right)=\frac{F_{1}}{F_{0}}\times 100$, where *F*_1_ and *F*_0_ are the final values obtained after and before treatment with the bioflocculant and nanoparticles, respectively.

### 2.9. Experimental, Software, and Statistical Analysis

All data was collected in triplicates and the error bars in the Figures show the standard deviations of the data. Data were subjected to one-way analysis of variances (ANOVA) using Graph Pad Prism™ 6.1. A significant level of p < 0.05 was used.

## 3. Results

### 3.1. FT-IT Spectra of the Bioflocculant and Iron Nanoparticles

**Figure 1:**
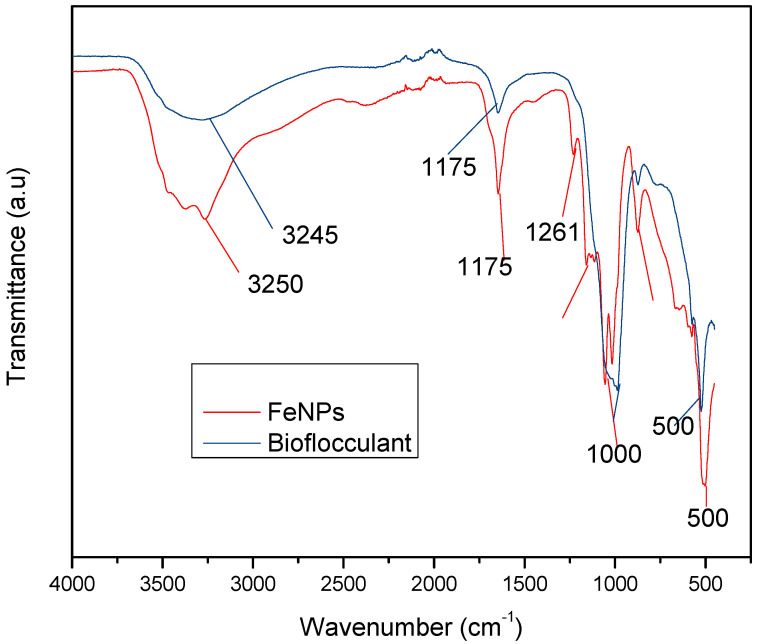
Fourier transform-infrared (FT-IR) spectra of the bioflocculant and iron nanoparticles.

Figure 1 below represents the functional groups present in the bioflocculant and iron nanoparticles. The peak at 3245 cm^−1^ (bioflocculant) and 3250 cm^−1^ (iron nanoparticles) indicates the presence of hydroxyl (–OH) and amine (–NH_2_) functional groups in the sample.

### 3.2. The SEM Morphology of the Bioflocculant and Iron Nanoparticles

**Figure 2:**
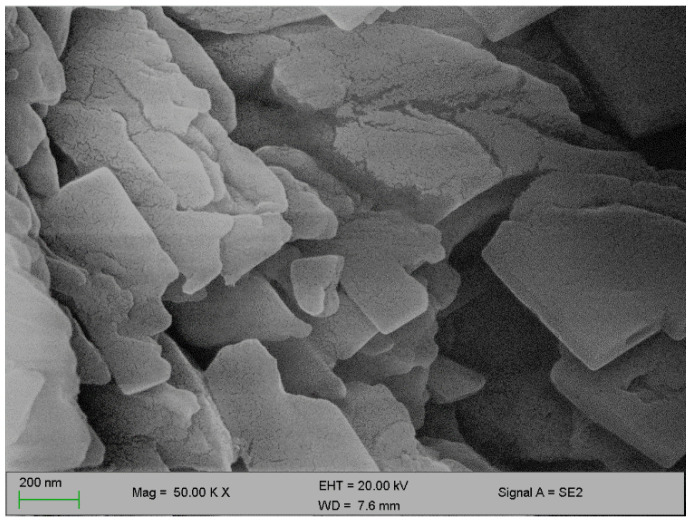
SEM surface morphology of the bioflocculant.

**Figure 3:**
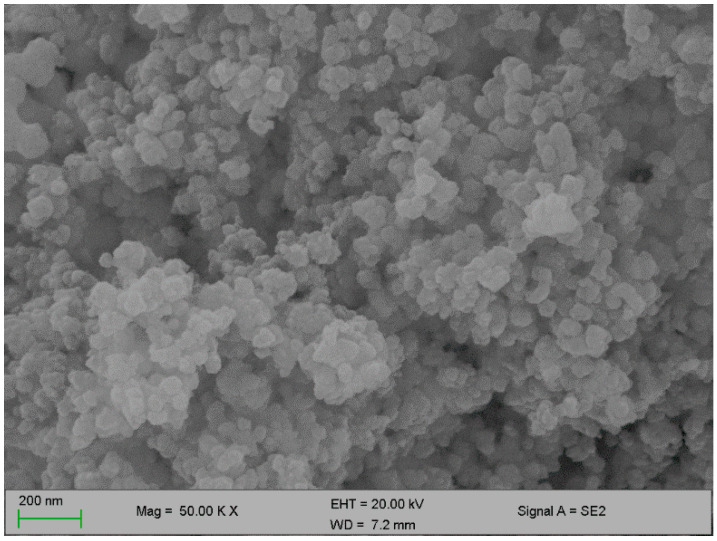
SEM surface morphology of the iron nanoparticles.

Figure 2 and Figure 3 below represent the surface morphology of the bioflocculant and iron nanoparticles, respectively. The bioflocculant has the crystal-like morphology while the nanoparticles seem to have granular-like shapes.

### 3.3. Elemental Composition of the Bioflocculant and Iron Nanoparticles

In Table 1 below, elements such as O, C, Mg, P, K, Ca, Fe, and Cu are present in the bioflocculant and iron nanoparticle samples. From both samples, oxygen and carbon account for over 50%, while iron and copper were only found to be present in the iron nanoparticles alone and absent in the bioflocculant.

### 3.4. Dosage Concentration Effect on Flocculation

**Figure 4:**
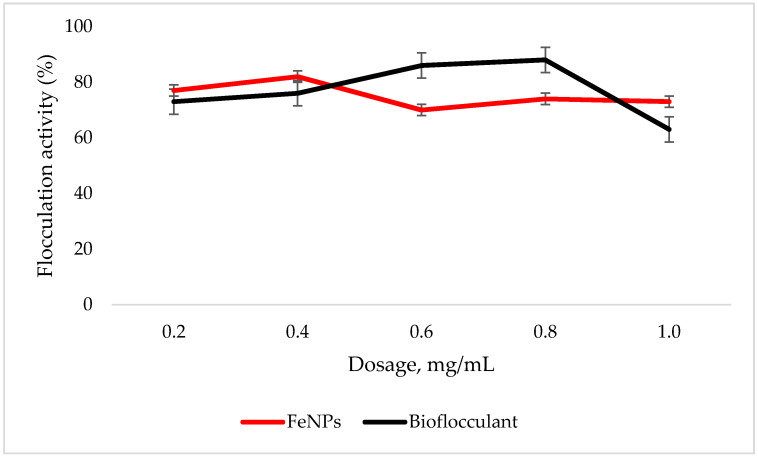
Dosage effect on flocculation activity. Error bars denote statistical significance at (p < 0.05).

An adequate dosage is required for an efficient flocculation process. Fe nanoparticles showed the optimum flocculation activity (FA) at 0.4 mg/mL dosage as opposed to that of the bioflocculant, which displayed the highest flocculation activity at 0.8 mg/mL (Figure 4). The optimum dosage for each flocculant was then used for subsequent experiments.

### 3.5. Temperature Effect on Flocculation Activity

**Figure 5:**
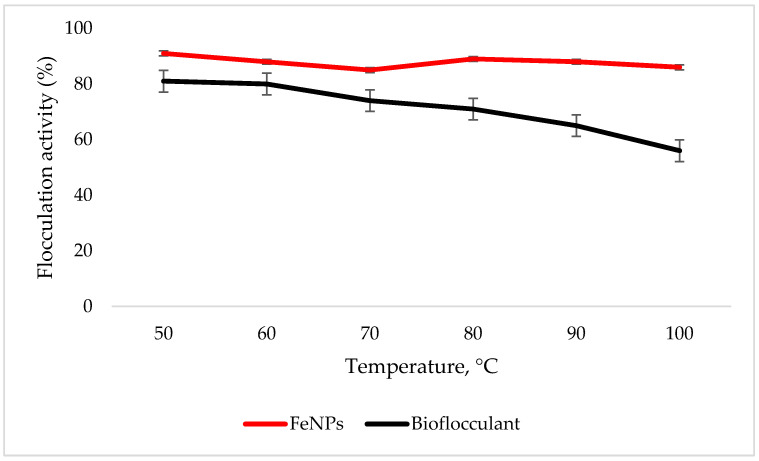
Temperature effect on flocculation activity. Error bars denote statistical significance at (p < 0.05).

The FeNPs are more thermostable compared to the bioflocculant, as the flocculation activity is above 86% at 100 °C, while the significant drop in flocculation activity is observed with the increased temperature in the bioflocculant (Figure 5).

### 3.6. Effect of pH on Flocculation Activity

**Figure 6:**
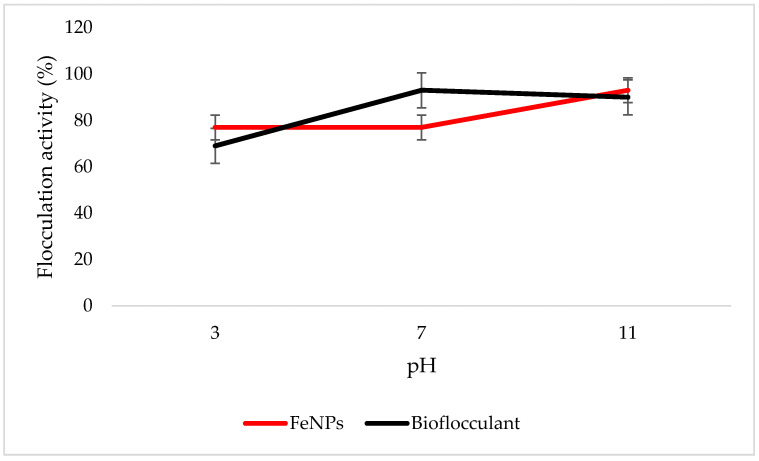
pH effect on flocculation activity. Error bars denote statistical significance at (p < 0.05).

Both the FeNPs and bioflocculant flocculate well in alkaline conditions, with FeNPs having the optimum flocculation activity at a pH of 11, while that of the bioflocculant is at a pH of 7 (Figure 6).

### 3.7. Effect of Metal Ions on Flocculation Activity

The nanoparticles and bioflocculant flocculated poorly when the cation was not added, with 49% and 46% flocculation activity, respectively (Table 2).

### 3.8. The Removal of COD and BOD

Table 3 represents removal efficiency by FeCl_3_, FeNPs, and the bioflocculant; Fe nanoparticles were the most effective in reducing both COD and BOD compared to the other two flocculants.

### 3.9. Evaluation of Cytotoxicity of the FeNPs and Bioflocculant

**Figure 7:**
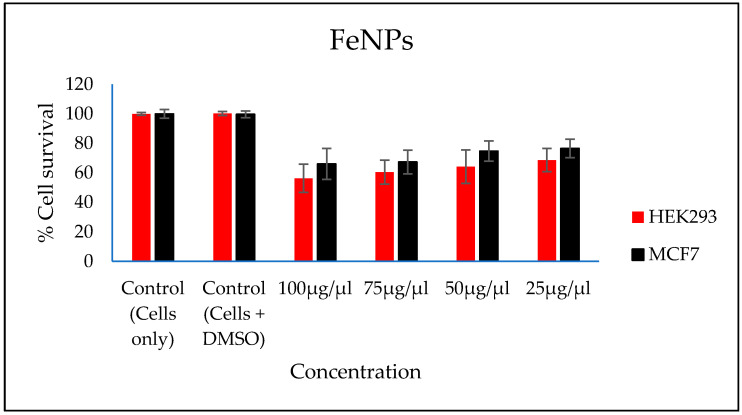
In vitro cytotoxicity effect of FeNPs nanoparticles on HEK293 and MCF7 cells.

**Figure 8:**
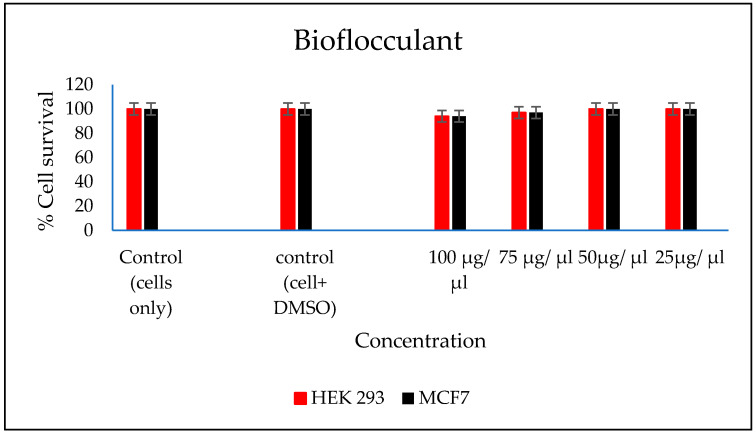
In vitro cytotoxicity effect of bioflocculant nanoparticles on HEK293 and MCF7 cells.

In vitro cytotoxicity of both the FeNPs and bioflocculant were evaluated and the FeNPs were found nontoxic at low concentrations and the bioflocculant was nontoxic at all concentrations (Figure 7 and Figure 8).

## 4. Discussion

The functional groups present in the molecular chains of the bioflocculant facilitate the binding capability of the bioflocculants [25]. The presence of the –OH group plays the significant role in reducing and stabilizing nanoparticles during synthesis [26]. Thermostability of the nanoparticles when subjected to heat further confirm the presence of the hydroxyl group. The flocculation process is influenced by the surface morphology of the flocculant and accounts for the effectiveness or poor efficiency of the flocculant [20]. In Figure 3, the crystal-like and granular morphology is observed. The change in the bioflocculant structure is the indication of the formation of nanoparticles in the synthesis. Furthermore, it can be noted that the nanoparticles have more surface area for pollutants absorption. Therefore, it can be deduced that the synthesis of nanoparticles does not only modify the surface structure, but it also increases the surface area on nanoparticles for particles flocculation and pollutants removal in water. The flexibility and stability of flocculants is brought about by the different elements present in the sample. In Table 1, elements such as O and C were found in the bioflocculant sample and account for a major percentage, as these elements form the backbone structure of the biomolecule. Furthermore, Mg, P, K, and Ca account for the production media that were used for the bioflocculant production. Similarly, the as-synthesized iron nanoparticles also had O and C, which account for 60.33%, and Fe was found to be the second highest present element at 17.31%, which indicates that the nanoparticles synthesis was successful. The copper grid, which was used during analysis, could account for 0.30% Cu present in the sample.

To effectively neutralize some of the negative charges on colloidal particles, an adequate dosage is required; if the dosage is insufficient, poor flocculation results [27]. Contrary to this, excess dosage may increase the viscosity, which results in poor flocculation activity [28]. As illustrated in Figure 4, the optimum flocculation activity was achieved at 0.4 mg/mL and 0.8 mg/mL for the nanoparticles and bioflocculant, respectively. An increase in flocculation activity was observed between 02–04 mg/mL for FeNPs, however, with the increase in dosage concentration to 0.6–1.0 mg/mL, the flocculation activity dropped a little and it remained consistent throughout. This could be due to the competition and repulsion of negatively charged kaolin particles, which in turn block binding sites. The low flocculation activity for the bioflocculant at 0.2–0.6 mg/mL may be due to the fact that low dosage did not permit bridging phenomena to occur effectively [11]. Both the bioflocculant and the FeNPs were subjected to different temperatures (50–100 °C) for 30 min in a water bath. As depicted in Figure 5, higher flocculation activity was observed at 50 °C with 91 and 81% for the nanoparticles and bioflocculant, respectively. The increase in temperature did not affect the flocculation process of the as-synthesized nanoparticles. The flocculation activity remained above 86%, suggesting that the nanoparticles are thermostable. The results are comparable with those of other studies [13,27,29], where heat could not affect the effectiveness of bioflocculants, indicating their thermostability. This could be attributed to the presence of the -OH group as indicated in Figure 1 above. The results are comparable with that of Sekelwa, et al. [30], where the presence of hydroxyl groups, evidenced by the IR spectra within the polymer, favored the possibility of hydrogen bonding with one or more water molecules.

Key factors that influence the flocculation process include pH. Flocculation activity may be affected by pH; it may alter flocculant status charge and surface characteristics of colloidal particles in suspension [27]. In Figure 6, the highest flocculation activity of 90% was achieved with FeNPs at a strong alkaline pH of 11. Nonetheless, the flocculation activity was still above 77% at a strong acidic pH of 3, suggesting that FeNPs can be applied in both acidic and alkaline conditions, but are most effective by using alkaline conditions. Contradictory to this, the flocculation activity of the bioflocculant was poor in acidic conditions with the optimum of 93% at a pH of 7. The poor performance in strong acidic conditions may be attributed to protein denaturation in the bioflocculant [11]. These findings suggest that the nanoparticles can be a suitable flocculant in coal mine waste, as the pH is mostly alkaline.

Residual negative net surface charge of the bioflocculant functional group is neutralized by cations, which in turn enhance the flocculation activity [31]. Various metal ion effects were evaluated on the as-synthesized nanoparticles and bioflocculant as shown in Table 2. The highest flocculation activity of 85% was observed when a trivalent cation (Fe^3+^) was used as an enhancing metal ion. However, both the monovalent and divalent cations could still have enhanced the flocculation activity with the flocculation activity above 70%. Contrary to this, the nanoparticles flocculate poorly without the presence of the cation, suggesting that they are cation-dependent. In the bioflocculant, both the monovalent and divalent were found to be most effective, with Li^+^ being the highest flocculation activity at 75%. The least flocculation activity was observed when the trivalent cation (Fe^3+^) was used. This conflicts the findings that suggest monovalent cations reduce the strength of the bonds and results in loose flocs, thus producing poor flocculation activity [32].

The higher amount of both COD and BOD is not good for the aquatic ecosystem. This condition results in the decrease of the amount of dissolved oxygen (DO), which in turn results in anaerobic conditions that are detrimental to higher aquatic life. Furthermore, a high amount of BOD in water signifies a high amount of nutrients, which may result in an algal bloom. In Table 3, different wastewaters were used to evaluate the effectiveness of FeNPs in comparison to a bioflocculant. Samples were analysed using a UV-Vis spectrophotometer Spectroquant^®^ at 620 nm wavelength. The removal of COD and BOD was conducted using the 0.4 and 0.8 mg/mL for the FeNPs and bioflocculant, respectively, as these concentrations were found to be effective from optimization in Figure 4. The nanoparticles proved to be most effective when compared to both the bioflocculant and ferric chloride with BOD over 80%, while COD was 76% for coal mine wastewater and least effective on river water with just 48%. Contrary to this, the Actinomycete bioflocculant that was used in the wastewater treatment and removal of heavy metals by Agunbiade et al. performed below 70% for both COD and BOD removal efficacy [13]. The bioflocculant remained consistently poor in all the samples for BOD removal with just 50% efficacy. However, a remarkable improvement was observed in COD removal for coal mine wastewater by the bioflocculant with 72%, but it remained poor in the river water sample. Therefore, it can be deduced that FeNPs are a better flocculant compared to the bioflocculant and ferric chloride. Bioflocculants are generally nontoxic but they still need to be tested for biosafety reasons [33]. In Figure 7, nanoparticles were evaluated against human normal cells (HEK 293) and cancer cells (MCF7). As-synthesized nanoparticles are found to be nontoxic at low concentrations, as the cell survival was above 76% for both cells at 25–50 µL. With the increase in concentration, cell survival rates decrease, however, cell survival was still above 56%. It is therefore recommended that FeNPs should not be used at high concentrations, as it may result in cell toxicity. Contrarily, the bioflocculant proved to be nontoxic against both cells at the highest concentration of 100 µL, with the cell survival over 90%.

## 5. Conclusions

The sample as-synthesized nanoparticles and bioflocculant revealed the presence of the functional groups –OH and –NH_2_, respectively. SEM-EDX indicated a huge percentage of O and C wt.% in both samples. FeNPs are most effective at low concentrations while the bioflocculant works best when the dosage is increased to 0.8 mg/mL. FeNPs are effective in all pH conditions and temperature ranges, while the bioflocculant was only effective at lower temperatures and neutral in weak alkaline conditions. Nanoparticles could remove effectively both COD and BOD in all water samples, while the bioflocculant and ferric chloride were seen to be less effective. FeNPs are nontoxic only at lower concentrations, while the bioflocculant is nontoxic even at higher concentrations. Therefore, FeNPs can be recommended as an alternative flocculant provided a lower concentration is maintained. For future prospects, more characterization should be conducted (XPS) to ascertain the oxidation state of the synthesized material. In addition, more characterization is necessary to establish the mechanism behind the formation of nanoparticles.

## Figures and Tables

**Table 1:** Energy-dispersive X-ray analysis (EDX) of the bioflocculant and iron nanoparticles.

Elements	Sample	
	Bioflocculant (wt.%)	FeNPs (wt.%)
C	13.21	12.39
O	55.25	47.94
Mg	13.35	1.12
P	16.00	13.43
K	0.14	0.24
Ca	2.04	7.33
Fe	-	17.31
Cu	-	0.30
Total	100.00	100.00

**Table 2:** Cation effect on flocculation activity.

Cations	Flocculation Activity (%)	
	Bioflocculant	FeNPs
Control	49 ± 3.35	46 ± 2.03 ^b^
Fe^3+^	31 ± 3.15	85 ± 2.72 ^a^
Mg^2+^	63 ± 6.78	82 ± 1.53 ^a^
Ca^2+^	71 ± 5.42	82 ± 3.64 ^a^
Li^+^	75 ± 2.31	72 ± 1.15 ^a^
Na^+^	62 ± 7.28	72 ± 1.15 ^a^

Different letters (^a^ and ^b^) denote statistical significance at (p < 0.05).

**Table 3:** Chemical oxygen demand (COD) and biochemical oxygen demand (BOD) removal in wastewater by the bioflocculant and iron nanoparticles.

Flocculant	Types of Waste Water	Types of Pollutants in Water	Water Quality before Treatment (mg/L)	Water Quality after Treatment (mg/L)	Removal Efficiency (%)
FeNPs	Coal mine water	COD BOD	842 123.2	204 23	76 81
	Mzingazi river water	COD BOD	3.300 136	1.700 24	48 82
Bioflocculant	Coal mine water	COD BOD	842 123.2	208 77.88	72 59
	Mzingazi river water	COD BOD	3.300 136	1.68 72.08	51 53
